# Treatments after progression to first-line FOLFOXIRI and bevacizumab in metastatic colorectal cancer: a pooled analysis of TRIBE and TRIBE2 studies by GONO

**DOI:** 10.1038/s41416-020-01089-9

**Published:** 2020-10-07

**Authors:** Daniele Rossini, Sara Lonardi, Carlotta Antoniotti, Daniele Santini, Gianluca Tomasello, Paola Ermacora, Marco Maria Germani, Francesca Bergamo, Vincenzo Ricci, Salvatore Caponnetto, Roberto Moretto, Alberto Zaniboni, Filippo Pietrantonio, Angela Buonadonna, Giuliana Ritorto, Gianluca Masi, Tiziana Pia Latiano, Stefania Rapisardi, Alfredo Falcone, Chiara Cremolini

**Affiliations:** 1 Department of Translational Research and New Technologies in Medicine and Surgery, University of Pisa, Pisa, Italy; 2 Unit of Medical Oncology 2, Azienda Ospedaliero-Universitaria Pisana, Pisa, Italy; 3 Unit of Medical Oncology 1, Department of Clinical and Experimental Oncology, Veneto Institute of Oncology, IOV - IRCSS, Via Gattamelata 64, 35128 Padova, Italy; 4 Department of Medical Oncology, Campus Bio-Medico—University of Rome, Via Álvaro del Portillo 200, 00128 Rome, Italy; 5 Oncology Unit, Oncology Department, ASST of Cremona, Viale Concordia 1, 26100 Cremona, Italy; 6 Department of Oncology, ASUFC University Hospital, Udine, Via Pozzuolo 330, 33100 Udine, Italy; 7 Medical Oncology and Laboratory of Translational Oncology, Oncology Department, S. Croce and Carle Teaching Hospital Cuneo, Via Michele Coppino 26, 12100 Cuneo, Italy; 8 Department of Radiological Science, Oncology And Pathology, Policlinico Umberto I, “Sapienza” University of Rome, Viale del Policlinico 155, 00161 Rome, Italy; 9 Medical Oncology Unit, Poliambulanza Foundation, Via Bissolati 57, 25124 Brescia, Italy; 10 Medical Oncology Department, Fondazione IRCSS Istituto Nazionale dei Tumori, Via Venezian 1, 20133 Milan, Italy; 11 Department of Oncology and Hemato-oncology, University of Milan, Via Festa del Perdono 7, 20122 Milan, Italy; 12 Department of Clinical Oncology, Centro di Riferimento Oncologico (CRO) IRCCS, Via Franco Gallini 2, 33081 Aviano, Italy; 13 Ssd Colorectal Cancer Unit Dipartimento Di Oncologia, AOU Città della Salute e della Scienza di Torino, Corso Bramante 88, 10126 Turin, Italy; 14 Oncology Unit, IRCCS Casa Sollievo della Sofferenza, Viale Cappuccini 1, 71013 San Giovanni Rotondo, Italy; 15 Oncology Unit, ARNAS Garibaldi Catania, Piazza Santa Maria di Gesù 5, 95100 Catania, Italy

**Keywords:** Colorectal cancer, Chemotherapy

## Abstract

**Background:**

FOLFOXIRI/bevacizumab (bev) is a first-line regimen of proven activity and efficacy in metastatic colorectal cancer. The upfront exposure to three cytotoxics raises concerns about the efficacy of treatments after progression.

**Methods:**

We performed a pooled analysis of treatments after progression to upfront FOLFOXIRI/bev in patients enrolled in two randomised Phase 3 studies (TRIBE and TRIBE2) that compared FOLFOXIRI/bev to doublets (FOLFOX or FOLFIRI)/bev. Response rate, progression-free survival (2nd PFS) and overall survival (2nd OS) during treatments after progression were assessed. The RECIST response in first line and the oxaliplatin and irinotecan-free interval (OIFI) were investigated as potential predictors of benefit from FOLFOXIRI ± bev reintroduction.

**Results:**

Longer 2nd PFS was reported in patients receiving FOLFOXIRI ± bev reintroduction compared to doublets ± bev or other treatments (6.1 versus 4.4 and 3.9 months, respectively, *P* = 0.013), and seems limited to patients achieving a response during first line (6.9 versus 4.2 and 4.7 months, respectively, *P* = 0.005) and an OIFI ≥ 4 months (7.2 versus 6.5 and 4.6 months, respectively, *P* = 0.045).

**Conclusions:**

First-line FOLFOXIRI/bev does not impair the administration of effective second-line therapies. First-line response and longer OIFI seem associated with improved response and 2nd PFS from FOLFOXIRI ± bev reintroduction, without impacting 2nd OS.

## Background

FOLFOXIRI (5-fluorouracil, L-leucovorin, oxaliplatin and irinotecan)/bevacizumab (bev) is regarded by main guidelines as an efficacious first-line therapeutic option for selected patients with metastatic colorectal cancer (mCRC),^1,2^ according to the results of several randomised trials.^3–7^

Recently, the Phase 3 TRIBE2 (NCT02339116) trial confirmed the superiority of the upfront exposure to FOLFOXIRI/bev followed by the reintroduction of the same regimen after first disease progression, as compared with the pre-planned sequential administration of the three cytotoxics across two subsequent lines of therapy (FOLFOX (5-fluorouracil, L-leucovorin and oxaliplatin)/bev followed by FOLFIRI (5-fluorouracil, L-leucovorin and irinotecan)/bev). In addition, the administration of triplet chemotherapy in first line did not prevent the administration of second-line treatment in the majority of enrolled patients. Indeed, the 80% of patients progressed after first-line FOLFOXIRI/bev received second-line therapy, consisting again in FOLFOXIRI±bev in the 69% of cases.^8^ Remarkably, also in the previous TRIBE trial that compared FOLFOXIRI/bev with FOLFIRI/bev as upfront treatment, where the choice of the treatment after progression was left at investigator’s choice, further therapy was administered to the majority of patients progressed after first line (80%).^3^

In spite of the survival advantage demonstrated with the triplet in both trials, supporting the long-term benefit of the upfront intensified strategy, concerns still exist about the most appropriate treatment to administer after the failure of FOLFOXIRI/bev. Options include the same triplet regimen and bev, doublet chemotherapy and an anti-angiogenic agent, anti-EGFR (Epidermal Growth Factor Receptor)-based regimens only in the case of *RAS* wild-type tumours,^1,2^ or later lines options (regorafenib or trifluridine/tipiracil) in the case of clearly refractory disease. Therefore, an optimal selection of the patients that could gain benefit from the reintroduction of the triplet, compared to a different strategy, is a challenge in the choice of the treatment after progression. In this setting, a retrospective pooled analysis of the OPTIMOX-1 and OPTIMOX-2 trials that evaluated 5-fluorouracil-based maintenance or chemoholidays following 6 months of upfront therapy with FOLFOX, demonstrated that patients with an oxaliplatin-free interval lasting at least 6 months were those who reported better outcomes following the reintroduction of oxaliplatin.^9^

Drawing from these considerations, we focused on therapies administered after first-line progression to patients treated with FOLFOXIRI/bev in the TRIBE and TRIBE2 studies and analysed their outcomes. In addition, with the aim of identifying predictors of benefit from the reintroduction of FOLFOXIRI/bev, we explored the association of clinical parameters of benefit from the first-line therapy, including the oxaliplatin and irinotecan-free interval (OIFI), with the efficacy of treatments after progression.

## Methods

### Study design

TRIBE (NCT00719797) and TRIBE2 (NCT02339116) are two Phase 3 randomised, open-label, multicentre trials that involved 1187 unresectable previously untreated mCRC patients. As previously detailed,^3,8^ in the TRIBE study, patients were randomised in a 1:1 ratio to receive up to 12 cycles of FOLFIRI/bev or FOLFOXIRI/bev followed by maintenance with 5-fluorouracil plus bev until disease progression, unacceptable adverse events, or consent withdrawal in both arms. Treatments after progression were left at investigators’ choice.^3^ In the TRIBE2 study, patients were randomised in a 1:1 ratio to FOLFOX/bev followed by FOLFIRI/bev after disease progression or FOLFOXIRI/bev followed by the reintroduction of the same agents after disease progression; all treatments were administrated up to eight cycles followed by 5-fluorouracil plus bev maintenance until disease progression, unacceptable adverse events or consent withdrawal.^8^

The present analysis included all patients enrolled in the TRIBE and TRIBE2 studies that progressed to upfront FOLFOXIRI/bev.

### Definition of endpoints

Treatments received after the first evidence of disease progression were collected. Overall response rate (ORR) was defined as the proportion of patients achieving partial or complete response during the treatment according to RECIST criteria version 1.0 and version 1.1 in TRIBE and TRIBE2 trials, respectively; 2nd progression-free survival (2nd PFS) was defined as the time from the beginning of the second-line treatment to the evidence of disease progression or death, whichever occurred first; 2nd overall survival (2nd OS) was defined as the time from the beginning of second-line treatment to death.

We investigated the association of RECIST response during the first-line therapy (CR + PR versus SD + PD) and of the oxaliplatin and irinotecan-free interval (OIFI), defined as the time from the last simultaneous administration of oxaliplatin and irinotecan in first line to disease progression (≥ versus <4 months), with clinical outcome during treatments after progression.

### Statistics

The chi-square test and Kruskall–Wallis test were used when appropriate, to compare clinical and biological features, and ORR between groups. Second PFS and 2nd OS were determined according to the Kaplan–Meier estimates method, and survival curves were compared using the log-rank test. Odds ratios (OR) and 95% confidence intervals (CI) were estimated with a logistic regression model, while hazard ratios (HR) and 95% CI were estimated with a Cox proportional hazard model.

All statistical tests were two-sided, and *P* values ≤0.05 were deemed significant. Statistical analyses were performed using SAS version 9.4 (SAS Institute, Inc., Cary, NC). The data cut-off for the present analysis was July 31, 2014 and July 30, 2019 for TRIBE and TRIBE2, respectively.

## Results

**Fig. 1:**
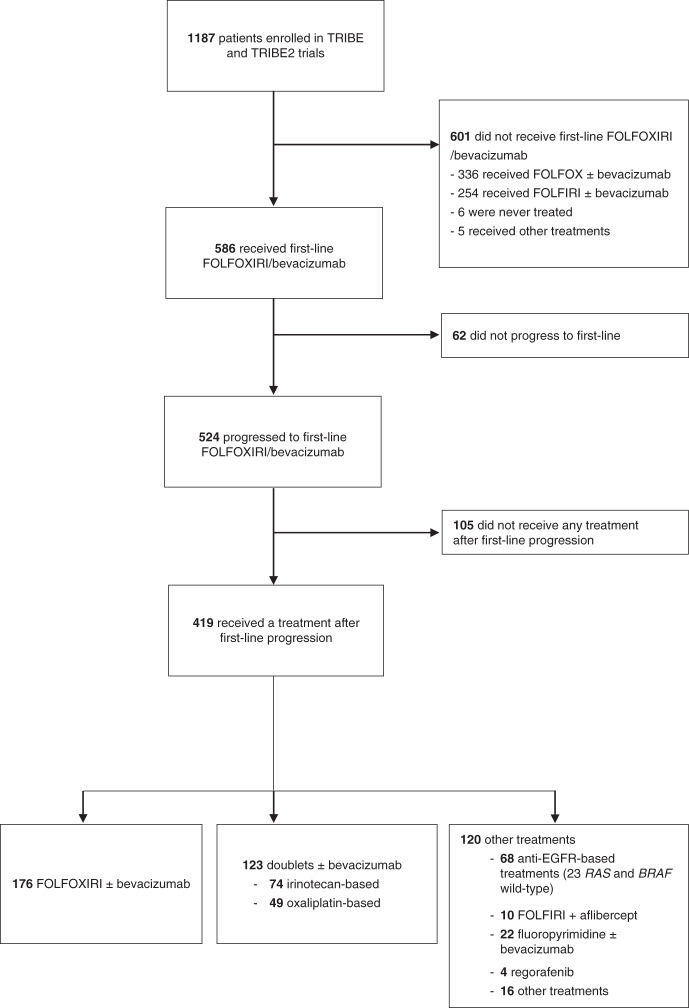
TRIBE and TRIBE2 pooled-analysis flowchart. EGFR: Epidermal Growth Factor Receptor.

Among 1187 patients enrolled in TRIBE and TRIBE2 studies, 586 received FOLFOXIRI/bev as upfront treatment and after a median follow up of 43.2 months, 524 of them experienced disease progression. In total, 419 (80%) patients received treatment after progression. The majority (70%) of those who did not receive any further treatment died within 3 months after progression. FOLFOXIRI ± bev was administered in 176 (42%) cases, while 123 patients (29%) received a doublet (irinotecan- or oxaliplatin-based in 74 (60%) and 49 (40%) cases, respectively) ± bev. The remaining 120 patients (29%) received other treatments, including anti-EGFR-based regimens in 68 cases (57%). Among patients receiving anti-EGFR-based therapies, 23 (34%) had a *RAS* and *BRAF* wild-type tumour, while a *RAS* or *BRAF* mutation was found in 16 (23%) and 6 (9%) cases, respectively. *RAS* and *BRAF* mutational status were not assessed in the remaining 23 (34%) patients (Fig. 1).

Table 1 summarises patients’ characteristics according to their treatment after progression. Therapies other than FOLFOXIRI ± bev or doublets ± bev were more frequently chosen for patients who achieved less clinical benefit from first-line FOLFOXIRI/bev (i.e., those who had not achieved RECIST response, and with an OIFI < 4 months).

**Table 1:** Patients’ characteristics.

Characteristics	FOLFOXIRI ± bevacizumab, *N* = 176 (%)	Doublets ± bevacizumab, *N* = 123 (%)	Other treatments, *N* = 120 (%)	*P*
*Age (years)*				0.22
Median	61	63	60	
Interquantile range	55–69	54–68	53–70	
*Sex*				0.10
Male	97 (55)	79 (64)	61 (51)	
Female	79 (45)	44 (36)	59 (49)	
*Site of primary tumour*				0.45
Right	62 (35)	38 (31)	50 (42)	
Left	81 (46)	54 (44)	47 (39)	
Rectum	33 (19)	28 (23)	21 (17)	
NA	–	3 (2)	2 (2)	
*Mutational status*				0.22
All wt	35 (20)	21 (17)	28 (23)	
RAS mut	119 (68)	74 (60)	50 (42)	
BRAF mut	13 (7)	9 (8)	8 (7)	
NA	9 (5)	19 (15)	34 (28)	
*Resected primary tumour*				0.18
Yes	95 (54)	70 (57)	77 (64)	
No	81 (46)	53 (43)	43 (36)	
*Time to metastases*				0.48
Synchronous	155 (88)	104 (85)	100 (83)	
Metachronous	21 (12)	19 (15)	20 (17)	
*Number of metastatic sites*				0.70
>1	108 (61)	79 (64)	79 (66)	
1	68 (39)	43 (35)	41 (34)	
NA	–	1 (1)	-	
*ECOG PS*				0.33
0	148 (84)	102 (83)	93 (77)	
1–2	28 (16)	21 (17)	27 (23)	
*Locoregional treatments in first line with curative intent*				0.58
Yes	31 (18)	27 (22)	21 (17)	
No	145 (82)	96 (78)	99 (83)	
*First-line response*				**0.05**
CR or PR	123 (70)	86 (71)	69 (57)	
SD or PD	53 (30)	37 (29)	51 (43)	
*First-line PFS* *≥* *9 months*				**0.04**
Yes	122 (69)	95 (77)	75 (63)	
No	54 (31)	28 (23)	45 (37)	
*OIFI*				**0.002**
≥4 months	133 (76)	86 (71)	67 (56)	
<4 months	43 (24)	37 (29)	53 (44)	

*N* number, *P* chi-square or Kruskal-Wallis test when appropriate, bold indicates statistical significance (*p*-value < 0.05), *NA* not available, *CR* Complete response, *ECOG-PS* Eastern Cooperative Oncology Group Performance Status, *OIFI* oxalilplatin and irinotecan-free interval, *PD* progression disease, *PFS* progression-free survival, *PR* partial response, *SD* stable disease.

**Fig. 2:**
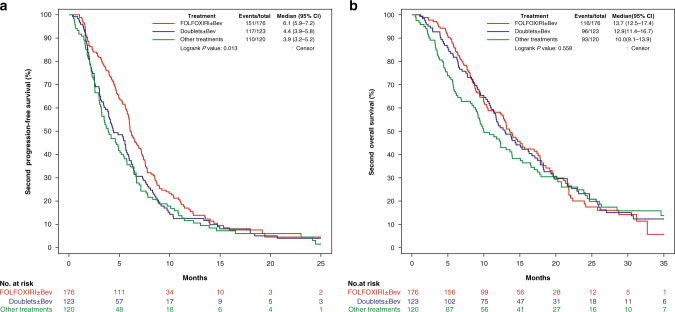
2nd PFS (a) and 2nd OS (b) according to treatment received after progression to first-line FOLFOXIRI/Bev. Bev bevacizumab, CI confidence interval.

Significant differences in terms of 2nd PFS (*P* = 0.013) were observed among the three groups (Fig. 2a). Patients treated with FOLFOXIRI ± bev showed longer 2nd PFS compared with those treated with doublets ± bev (median 2nd PFS: 6.1 versus 4.4 months, HR: 0.76 (95% CI: 0.60–0.97), *P* = 0.029) or other treatments (median 2nd PFS: 6.1 versus 3.9 months, HR: 0.71 (95% CI: 0.56–0.91), *P* = 0.007). Significant differences were also reported in terms of activity (*P* = 0.029): higher ORR was reported in favour of FOLFOXIRI ± bev compared with doublets ± bev (23% versus 11%, OR: 2.29 (95% CI: 1.18–4.42), *P* = 0.012), and a not significant difference compared with other treatments (23% versus 15%; OR: 1.67 (95% CI: 0.90–3.08), *P* = 0.10) was reported (Table 2). The 2nd OS did not differ (*P* = 0.558) among groups (Fig. 2b). In particular, no difference was observed between patients receiving FOLFOXIRI ± bev reintroduction and doublets ± bev (median 2nd OS: 13.7 versus 12.9 months, HR: 1.00 (95% CI: 0.76–1.31), *P* = 1.00) or other treatments (median 2nd OS: 13.7 versus 10.0 months, HR: 0.86 (95% CI: 0.65–1.14), *P* = 0.29).

**Table 2:** Overall response rate, 2nd progression-free survival and 2nd overall survival according to second-line treatments.

Patients receiving second-line treatment, *N* = 419								
	FOLFOXIRI ± bev, *N* = 176	Doublets ± bev, *N* = 123	HR/OR (95% CI)^a^	*P* value^a^	Other treatments, *N* = 120	HR/OR (95% CI)^b^	*P* value^b^	*P* value^c^
ORR (%)	23	11	2.29 (1.18–4.42)	**0.012**	15	1.67 (0.90–3.08)	0.10	**0.031**
2nd PFS (months)	6.1	4.4	0.76 (0.60–0.97)	**0.029**	3.9	0.71 (0.56–0.91)	**0.007**	**0.013**
2nd OS (months)	13.7	12.9	1.00 (0.76–1.31)	1.00	10	0.86 (0.65–1.14)	0.29	0.558

*2nd PFS* progression-free survival during second line, *2nd OS* overall survival during second line, *Bev* bevacizumab, *CI* confidence interval, *HR* hazard ratio, *ORR* overall response rate, *N* number, *OR* odds ratio, bold indicates statistical significance (*p*-value < 0.05).

^a^FOLFOXIRI ± bev versus doublets ± bev.

^b^FOLFOXIRI ± bev versus other treatments.

^c^FOLFOXIRI ± bev versus doublets ± bev versus other treatments.

As expected, patients achieving a response in first line had a higher chance to respond again to the treatment after progression (OR: 3.32, (95% CI: 1.69–6.54), *P* < 0.001) and to report longer 2nd PFS (5.9 versus 4.3 months, HR: 0.76 (95% CI: 0.62–0.95), *P* = 0.013).

**Fig. 3:**
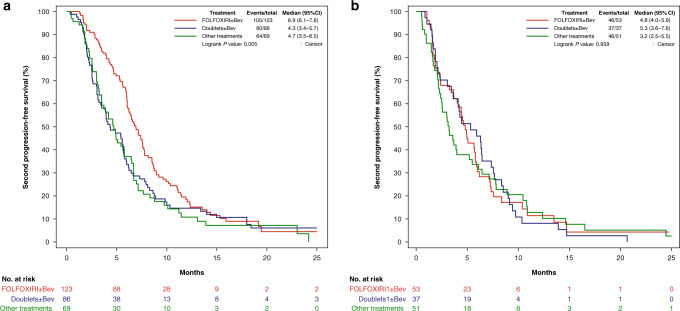
2nd PFS in patients achieving (a) or not (b) an objective response to first-line FOLFOXIRI/Bev according to treatment received after progression. Bev bevacizumab, CI confidence interval.

Among first-line responders, benefit from FOLFOXIRI ± bev reintroduction was reported in terms of both ORR and 2nd PFS compared to doublets ± bev (ORR 29% versus 12%, OR: 3.15 (95% CI: 1.46–6.76), *P* = 0.003; median 2nd PFS: 6.9 versus 4.3 months, HR: 0.68 (95% CI: 0.51–0.91), *P* = 0.010). A significant advantage in terms of 2nd PFS was also observed for FOLFOXIRI ± bev compared with other treatments (median 2nd PFS: 6.9 versus 4.7 months, HR: 0.63 (95% CI: 0.46–0.87), *P* = 0.004) (Fig. 3a). Conversely, no differences in treatment outcome were reported among patients who had not achieved response to first-line FOLFOXIRI/bev in terms of ORR (*P* = 0.699), 2nd PFS (*P* = 0.959) (Fig. 3b), or 2nd OS (*P* = 0.527) (Supplementary Table 1).

Patients with an OIFI ≥ 4 months showed longer 2nd PFS (median 2nd PFS: 6.1 versus 3.7 months, HR: 0.52 (95% CI: 0.42–0.65), *P* < 0.001) (Supplementary Fig. 1A) and 2nd OS (median 2nd OS: 16.5 versus 8.7 months, HR: 0.43 (95% CI: 0.34–0.55), *P* < 0.001) (Supplementary Fig. 1B) compared to patients with an OIFI < 4 months.

**Fig. 4:**
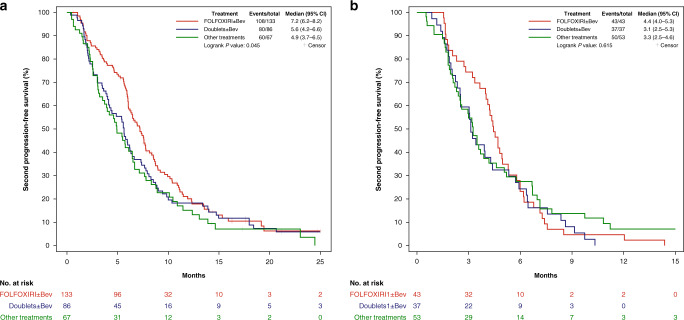
2nd PFS in patients achieving an OIFI ≥ 4 months (a) or <4 months (b) according to treatment received after progression to first-line FOLFOXIRI/Bev. Bev bevacizumab, CI confidence interval.

Among patients with an OIFI ≥ 4 months FOLFOXIRI ± bev reintroduction led to a positive trend towards better 2nd PFS when compared to doublets ± bev (median 2nd PFS: 7.2 versus 5.6 months, HR: 0.77 (95% CI: 0.58–1.04), *P* = 0.083), and to longer 2nd PFS compared to other treatments (median 2nd PFS: 7.2 versus 4.9 months, HR: 0.69 (95% CI: 0.50–0.95), *P* = 0.022) (Fig. 4a). No differences were observed in terms of ORR (*P* = 0.173) or 2nd OS (*P* = 0.916) (Supplementary Table 2). No differences in treatment outcome were reported among patients with an OIFI < 4 months, in terms of ORR (*P* = 0.270), 2nd PFS (*P* = 0.615) (Fig. 4b), or 2nd OS (*P* = 0.836).

## Discussion

Starting the therapeutic route of mCRC patients with the upfront exposure to the three active cytotoxics raised doubts about the efficacy and feasibility of treatments after progression. Two Phase 3 randomised trials demonstrated a survival advantage with first-line FOLFOXIRI/bev compared with doublets/bev thus showing that the benefit from the intensified upfront regimen was not limited to the first step of patients’ treatment but translated into a long-term effect. Moreover, the initial advantage was not subsequently lost, thus suggesting that treatments after progression could be still efficacious.^3,8^

In the present pooled analysis of the TRIBE and TRIBE2 study, we confirm the feasibility of treatments after progression to first-line FOLFOXIRI/bev showing that the 80% of patients progressed to the first-line therapy were then able to receive a subsequent systemic therapy. Though acknowledging all the limitations of cross-trials comparisons including the heterogeneity of different studies’ inclusion criteria, outcome results reported by patients receiving doublets + /− bev were similar to those of trials investigating the continuation of bev beyond progression or the efficacy of other anti-angiogenic agents combined with doublets in terms of OS and slightly worse in terms of PFS.^10–13^

Notably, the reintroduction of FOLFOXIRI ± bev was chosen in the 42% of patients receiving a treatment after progression. When interpreting this percentage, it should be mentioned that while the reintroduction strategy was prospectively planned in the TRIBE2 study, this was not recommended or suggested in the TRIBE trial, where treatments after progression were left at investigators’ choice. The reintroduction of FOLFOXIRI ± bev provided a significant 2nd PFS benefit compared to both doublets ± bev and other strategies, with consistent activity results.^3,8^

Reintroducing oxaliplatin after progression to a first-line oxaliplatin-based therapy is a valuable therapeutic option along the continuum of care of mCRC patients, as demonstrated by several retrospective and prospective analyses.^9,14,15^ This strategy is likely more efficacious for those patients who achieved higher benefit from the upfront therapy, with a longer interval between the last administration of oxaliplatin and the evidence of disease progression. The shorter duration of the induction therapy in TRIBE2 (4 months) compared with TRIBE (6 months) led to similar PFS results (median PFS: 12 months in both studies) and may have favourably affected the reintroduction rate, as a result of the lower incidence of serious neurotoxicity. In the targeted agents’ era, prolonging this time interval is made possible thanks to the efficacy of maintenance strategies able to delay disease progression. Evidence about the efficacy of reintroducing the triplet is currently much more limited.^16^

In order to identify patients more likely to achieve benefit from the reintroduction of the triplet ± bev, we investigated the association of parameters of benefit from first-line FOLFOXIRI/bev with the efficacy of treatments after progression. Patients who had achieved an objective response during the first-line therapy reported a significant advantage from the reintroduction of the triplet instead of other treatments after progression in terms of both objective responses, and 2nd PFS that was not observed among individuals who did not respond to the first-line therapy. Similarly, a trend towards longer 2nd PFS was observed in favour of the triplet ± bev in patients with a ≥ 4 months interval from the last administration of oxaliplatin and irinotecan to the first evidence of disease progression. Consistently with our results, in the pooled analysis of the OPTIMOX-1 and OPTIMOX-2 trials higher ORR and PFS were reported in the subgroup of patients receiving oxaliplatin reintroduction after two disease assessments by means of CT scan.^9^

The retrospective nature of our work prevents us from drawing conclusions about the different efficacy of triplet ± bev versus doublets ± bev versus other options as treatments after progression to upfront FOLFOXIRI/bev. In fact, the lack of randomisation does not allow to exclude that clinical outcomes reported with administered therapies might have been affected by unbalances in prognostic factors at the time of their beginning. In particular, alternative treatment strategies were chosen more frequently in patients who achieved less benefit from the upfront therapy probably as a result of more aggressive tumour biology or resistance to the upfront approach. To this regard, we were not able to assess the role of anti-EGFR-containing regimens in second line compared with other strategies in *RAS* and *BRAF* wild-type patients due to the limited sample size. In the PRODIGE-18 trial, favourable results were reported with the continuation of bev in combination with a switched chemotherapy as second-line therapy of *KRAS* wild-type patients treated with first-line chemotherapy plus bev compared with second-line chemotherapy plus anti-EGFRs.^17^

At the same time, the association of the second-line treatment outcome with the benefit from the upfront regimen may be explained by the better prognosis of those tumours but also by the effect of the intensified upfront therapy in modifying tumour biology. In fact, it is well established that achieving response with the first-line therapy positively affects patients’ long-term outcome independently of prognostic features at baseline.

Finally, we were not able to assess the safety profile of therapies after progression since adverse events that occurred were not collected in the TRIBE study. TRIBE2 showed no increased toxicity with FOLFOXIRI/bev compared with FOLFIRI/bev after progression with the exception of a higher incidence of neuropathy in a population clinically selected according to general conditions but also to the previous tolerance during the first-line therapy. A clear limitation is the lack of quality of life data from both trials.^3,8^

In conclusion, upfront FOLFOXIRI/bev does not impair the efficacy of treatments after progression. The reintroduction of FOLFOXIRI ± bev may provide an incremental benefit in ORR and 2nd PFS compared to other options with no significant OS advantage, and should be considered especially in fit patients, with good treatment tolerance, no moderate/severe residual neurotoxicity who experienced objective response during the first-line therapy and with a ≥4 months OIFI.

Oppositely, alternative treatment strategies should be considered, if available, in patients with limited benefit from the upfront therapy, including anti-EGFR-based regimens in *RAS*/*BRAF* wild-type tumours;^18^ BRAF and EGFR inhibitors in BRAF V600E mutant;^19^ FOLFIRI plus aflibercept or ramucirumab,^12,13^ trifluridine/tipiracil or regorafenib in *RAS* mutant.^20–24^

## Supplementary information

Supplementary Files

## Data Availability

Datasets supporting the results of this work are available to editors, referees and readers promptly upon request.
