# Supplementary material for: Treatments after progression to first-line FOLFOXIRI and bevacizumab in metastatic colorectal cancer: a pooled analysis of TRIBE and TRIBE2 studies by GONO
Source: Br J Cancer. 2020 Oct 7;124(1):183–90. doi: 10.1038/s41416-020-01089-9 (PMC7782547; doi:10.1038/s41416-020-01089-9)
Supplement: Supplementary file 1 — Supplementary Files [file 41416_2020_1089_MOESM1_ESM.docx]

**Supplementary Tables**

**Supplementary Table 1**

| **Overall response rate, 2^nd^ progression-free survival and 2^nd^ overall survival according to second-line treatments** | | | | | | | | |
| --- | --- | --- | --- | --- | --- | --- | --- | --- |
| **Patients receiving second-line treatment, N=419** | | | | | | | | |
| **PR or CR to first-line, N=278** | | | | | | | | |
|  | **FOLFOXIRI ± bev,**  **N=123** | **Doublets ± bev,**  **N=86** | **HR/OR**  **(95% C.I.)** | **p value*** | **Other**  **treatments,**  **N=69** | **HR/OR**  **(95% C.I.)** | **p value**** | **p value***** |
| **ORR (%)** | 29 | 12 | 3.15  (1.46-6.76) | **0.003** | 22 | 1.49  (0.75-2.97) | 0.259 | **0.013** |
| **2^nd^ PFS (months)** | 6.9 | 4.3 | 0.68  (0.51-0.91) | **0.010** | 4.7 | 0.63  (0.46-0.87) | **0.004** | **0.005** |
| **2^nd^ OS (months)** | 17.4 | 13.9 | 0.90  (0.64-1.26) | 0.548 | 13.6 | 1.00  (0.63-1.29) | 0.568 | 0.745 |
| **SD or PD to first-line, N=141** | | | | | | | | |
|  | **FOLFOXIRI ± bev,**  **N=53** | **Doublets ± bev,**  **N=37** | **HR/OR**  **(95% C.I.)** | **p value*** | **Other**  **treatments,**  **N=51** | **HR/OR**  **(95% C.I.)** | **p value**** | **p value***** |
| **ORR (%)** | 8 | 11 | 0.67  (0.16-2.88) | 0.594 | 6 | 1.31  (0.28-6.15) | 0.735 | 0.699 |
| **2^nd^ PFS (months)** | 4.8 | 5.3 | 1.05  (0.68-1.62) | 0.840 | 3.2 | 0.91  (0.60-1.37) | 0.644 | 0.959 |
| **2^nd^ OS (months)** | 8.8 | 11.6 | 1.30  (0.81-2.08) | 0.278 | 8.6 | 0.94  (0.61-1.46) | 0.796 | 0.527 |

2^nd^PFS: progression-free survival during second-line; 2^nd^OS: overall survival during second-line; Bev: bevacizumab; CI: Confidence Interval; HR: Hazard Ratio; ORR: overall response rate; N=number; OR=odds ratio.

* FOLFOXIRI ± bevacizumab *versus* doublets ± bevacizumab

** FOLFOXIRI ± bevacizumab *versus* other treatments

*** FOLFOXIRI ± bevacizumab *versus* doublets ± bevacizumab *versus* other treatments

**Supplementary Table 2**

| **Overall response rate, 2^nd^ progression-free survival and 2^nd^ overall survival according to second-line treatments** | | | | | | | | |
| --- | --- | --- | --- | --- | --- | --- | --- | --- |
| **Patients receiving second-line treatment, N=419** | | | | | | | | |
| **OIFI ≥ 4 months, N=286** | | | | | | | | |
|  | **FOLFOXIRI ± bev,**  **N=133** | **Doublets ± bev,**  **N=86** | **HR and OR (95% C.I.)** | **p value*** | **Other**  **treatments,**  **N=67** | **HR and OR**  **(95% C.I.)** | **p value**** | **p value***** |
| **ORR (%)** | 26 | 15 | 1.93  (0.95-3.91) | 0.066 | 20 | 1.43  (0.69-2.93) | 0.941 | 0.173 |
| **2^nd^ PFS (months)** | 7.2 | 5.6 | 0.77  (0.58-1.04) | 0.083 | 4.9 | 0.69  (0.50-0.95) | **0.022** | **0.045** |
| **2^nd^ OS (months)** | 17.4 | 16.3 | 1.09  (0.77-1.55) | 0.608 | 16.5 | 1.07  (0.73-1.56) | 0.742 | 0.916 |
| **OIFI < 4 months, N=153** | | | | | | | | |
|  | **FOLFOXIRI ± bev,**  **N=43** | **Doublets ± bev,**  **N=37** | **HR and OR**  **(95% C.I.)** | **p value*** | **Other**  **treatments,**  **N=53** | **HR and OR**  **(95% C.I.)** | **p value**** | **p value***** |
| **ORR (%)** | 14 | 3 | 5.83  (0.67-50.93) | 0.076 | 9 | 1.56  (0.44-5.50) | 0.489 | 0.270 |
| **2^nd^ PFS (months)** | 4.4 | 3.1 | 0.84  (0.54-1.32) | 0.460 | 3.3 | 0.99  (0.65-1.50) | 0.948 | 0.615 |
| **2^nd^ OS (months)** | 8.8 | 9.1 | 0.88  (0.56-1.39) | 0.585 | 6.8 | 0.88  (0.58-1.34) | 0.551 | 0.836 |

2^nd^PFS: progression-free survival during second-line; 2^nd^OS: overall survival during second-line; Bev: bevacizumab; CI: Confidence Interval; HR: Hazard Ratio; OIFI: oxaliplatin irinotecan free interval; N=number; OR=odds ratio.

* FOLFOXIRI ± bevacizumab *versus* doublets ± bevacizumab

** FOLFOXIRI ± bevacizumab *versus* other treatments

*** FOLFOXIRI ± bevacizumab *versus* doublets ± bevacizumab *versus* other treatments

**Supplementary Figure 1**


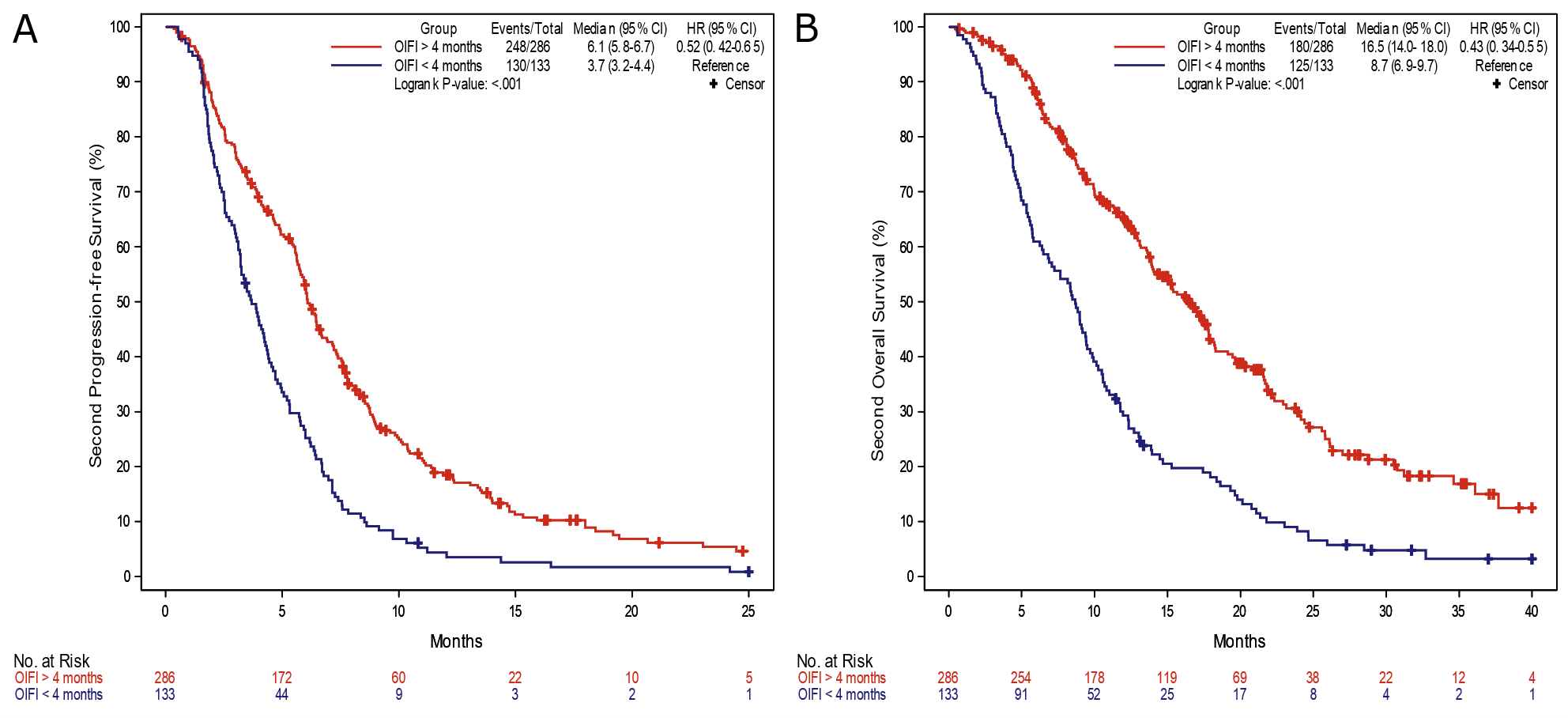


**Supplementary Figure 1: 2^nd^ PFS according to OIFI.** CI: Confidence Interval; HR: Hazard Ratio; OIFI: oxaliplatin irinotecan free interval
